# siRNA-mediated silencing of integrin β3 expression inhibits the metastatic potential of B16 melanoma cells

**DOI:** 10.3892/or.2012.1963

**Published:** 2012-08-10

**Authors:** ANNA NASULEWICZ-GOLDEMAN, BARBARA USZCZYŃSKA, KATARZYNA SZCZAURSKA-NOWAK, JOANNA WIETRZYK

**Affiliations:** Department of Experimental Oncology, Institute of Immunology and Experimental Therapy, Polish Academy of Sciences, 12 R. Weigl Street, 53-114 Wroclaw, Poland

**Keywords:** integrins, metastasis, mouse melanoma, RNA interference, siRNA

## Abstract

Integrins comprise a large family of αβ heterodimeric cell-surface receptors that mediate diverse processes involved in cell-cell and cell-matrix interactions such as cellular adhesion and migration, cell survival and differentiation. It is now well documented that integrins play a crucial role in cancer metastasis and angiogenesis. The β3 integrins appear to have an important stimulatory role in tumour progression and metastasis and, thus, have been often proposed as potential targets for cancer diagnosis and therapy. In this study, we evaluated the *in vitro* and *in vivo* properties of B16 mouse melanoma cells with low expression of integrin β3. Proliferation rate, adhesive properties and the ability to migrate and metastasize were studied. Over 90% inhibition of integrin β3 expression was achieved as a result of the transfection with siRNA. No changes in the proliferation rate were observed in siRNA-transfected B16 cells; however, they showed impaired ability to bind to fibronectin. Moreover, inhibition of integrin β3 expression caused almost complete impairment of the ability of B16 cells to migrate through matrigel and metastasize. The mean number of lung metastatic colonies in mice inoculated intravenously with B16 expressing low levels of integrin β3 was decreased to 14 colonies compared to 101 in the control group. These results provide evidence for a direct role of integrin β3 in the adhesion, migration and metastasis processes of mouse melanoma cells and point to the potential therapeutic advantages of siRNAs.

## Introduction

Integrins comprise a large family of αβ heterodimeric cell-surface receptors that are expressed in a wide variety of cells. They mediate diverse processes and are involved in cell-cell and cell-matrix interactions such as cell adhesion and migration, cell survival and differentiation. It is now well documented that integrins play a crucial role in cancer progression, metastasis and neoangiogenesis.

There are two members of integrin β3 family: αvβ3 and αIIbβ3. αvβ3 integrin is strongly expressed on the surface of the smooth muscle cells, endothelial cells, monocytes and platelets. Dysregulation of β3 integrin expression is associated with the pathogenesis of several diseases, including cancer. Many invasive tumour cells, including melanoma show an overexpression of this integrin. There are also reports indicating the correlation between αvβ3 integrin expression and the stage of tumour progression (1–5). β3 integrins are also strongly involved in tumour-induced angiogenesis and have been described as pro-angiogenic factors (6,7). The role of αvβ3 integrin in tumour angiogenesis is related not to its expression by neoplastic cells, but rather to its expression by host endothelial cells (8). Moreover, it was proven that antagonists of αvβ3 inhibit angiogenic processes, including endothelial cell adhesion and migration, whereas factors, which increase αvβ3 integrin expression, induce angiogenesis (9,10).

αIIbβ3 integrin expression is limited mainly to platelets, megakaryocytes, human blood monocytes, granulocytes, and large granular lymphocytes (11). However, there is increasing evidence that αIIbβ3 integrin is also present in the tumour cells (3). Its expression is connected with tumour thickness, invasion abilities and metastatic potential of human and mouse melanomas (3,8). Various studies showed that αIIbβ3 is constitutively expressed at a high-affinity state and is highly involved in tumour cell adhesion and invasion (12).

αIIbβ3 integrin is also involved in tumour-induced platelet aggregation, which has been described as an important step of metastasis pathway. Tumour cells during migration in blood vessels can form complexes with platelets. This process, resulting from direct binding of platelets to tumour cells, is essential for metastasis (8,13).

The β3 integrins appear to have an important stimulatory role in tumour progression and metastasis and that is why β3 integrins have often been proposed as potential targets for cancer diagnostic and therapeutic approaches. Application of anti-integrin antibodies and RGD (Arg-Gly-Asp) related peptides have revealed promising effects in anticancer therapy (14–17). One of the most interesting integrin-targeting tools are short interfering RNAs (siRNAs).

In this study, the *in vitro* and *in vivo* properties of B16 mouse melanoma cells with lower expression of integrin β3 were evaluated. Proliferation rate, adhesive properties and the ability to migrate and metastasize were studied. In order to achieve cells with low expression of integrin β3, transfection with siRNA was employed. B16 cells that fail to express integrin β3 show impaired motility and ability to bind to extracellular matrix (ECM) proteins, and are unable to colonize lungs. These results provide supplementary data for a direct role of integrin β3 in the adhesion, migration and metastasis processes of mouse melanoma cells and prove that the silencing of integrin expression can be efficiently and selectively obtained using siRNAs.

## Materials and methods

### Cell culture

The mouse melanoma B16 cells were obtained from the American Type Culture Collection (Rockville, MD, USA) and maintained in the Cell Culture Collection of the Institute of Immunology and Experimental Therapy Polish Academy of Sciences (IIET, PASc), Wroclaw, Poland. Cells were cultured in RPMI medium supplemented with 4 mM L-glutamine, 4.5 g/l glucose, 1.5 g/l NaHCO_3_ (both from Sigma-Aldrich Chemie GmbH, Steinheim, Germany), 100 U/ml penicillin, 100 μg/ml streptomycin (both from Polfa Tarchomin S.A., Warsaw, Poland) and 10% FBS (Sigma-Aldrich Chemie GmbH).

### siRNA

The siRNAs (sense and antisense strands) were purchased from Qiagen (Qiagen Inc., Valencia, USA) and were diluted according to manufacturer’s instructions and then stored at −20°C. The following sequences were tested for their effectiveness in silencing integrin β3 expression: Sequence M1: sense r(GCCGUGAAUUGUACCUACA)dTdT, antisense r(UGUAGGUACAAUUCACGGC)dGdT; Sequence M2: sense r(CGGUGAGCUUUAGUAUCGA)dTdT, antisense r(UCGAUACUAAAGCUCACCG)dTdG. As a control, a negative siRNA, with no homology to mRNA databases was used (Silencer^®^ Negative Control #1 siRNA, Ambion).

*In vitro* transfections were performed using HiPerFect reagent (Qiagen Inc.) as recommended by the manufacturer. Cells were plated on a 24-well plate in 0.5 ml of medium RPMI-O-MEM without antibiotics and FBS (4×10^4^ cells per well). Shortly after plating, cells were transfected with 100 μl of the transfection mixture containing 5 or 25 nM of siRNA. Cells were washed 6 h after transfection and the procedure was repeated 48 h later.

### Integrin quantification

The expression of integrin β3 (CD61) (Becton Dickinson, San Jose, USA) was determined by flow cytometry. B16 cells (1×10^5^) were mixed with an appropriate volume of McAb solution (pre-chilled to 4°C). Cells were incubated for 30 min on an ice bath, and subsequently washed twice with PBS (supplemented with 2% fetal bovine serum). Cell surface fluorescence was measured using a FACS Calibur flow cytometer (Becton Dickinson). Damaged cells were labeled with propidium iodide solution to each test tube just before data acquisition. Data for damaged cells were not analyzed. Data analysis was performed using WinMDI 2.8 software.

### Semi-quantitative PCR

Total RNA extraction, DNA digestion and cDNA synthesis was performed with RNAlater RNA Stabilization Reagent™ (Qiagen Inc.) according to the manufacture’s procedure. PCR reaction was performed using the following primers: integrin β3: forward 5′TCAGATGCGCAAGCTTACTAGC3′, reverse 5′TCAGCACGTGTTTGTAGCCAA3′; GAPDH: forward: 5′ATGACATCAAGAAGGTGGTG3′, reverse: 5′CATACCAGGAAATGAGCTTG3′. PCR cycling conditions were 94°C for 30 sec, 55°C for 30 sec, and 72°C for 1 min, 35 cycles for integrin β3 expression and 25 cycles for GAPDH. PCR products were dissolved in 1.7% agarose gel with ethidium bromide.

### Antiproliferative assays

Cells were plated in 96-well plates (Sarstedt, Inc. Newton, NC, USA) at the density of 8×10^3^ cells per well in 100 μl of culture medium without FBS and antibiotics. After 24 h of incubation at standard conditions (37°C in humid atmosphere with 5% CO_2_), cells were treated with siRNA suspended in 100 μl of medium FBS and antibiotics-free. The cytotoxic assays were performed after 24, 48 and 72 h exposure of the cultured cells to varying concentrations siRNA, e.g. 1, 5 and 25 nM. The amount of HiPerFect was stable (3 μl per well). The SRB method was used as described by Skehan and coworkers (18). The optical densities of the samples were measured on a Multiskan RC photometer (Labsystems, Helsinki, Finland) at λ=540 nm.

### Adhesion assay

Flat-bottomed, 96-well plates were coated with fibrinogen (10 μg/ml suspended in 7.5% NaHCO_3_, Merck, Darmstadt, Germany) and blocked with 1% BSA (Sigma-Aldrich Chemie GmbH) in TSM buffer (20 mM Tris-HCl pH 8.0, 150 nM NaCl, 1 mM CaCl_2_, 2 mM MgCl_2_). Cells were suspended in 0.5% solution of BSA, added into plates in the amount of 2.5×10^4^ and incubated for 1 h at 37°C. Unbound cells were washed out twice with TSM buffer and dyed with 0.2% solution of crystalline violet in methanol. After 30 min of incubation at 4°C, cells were washed with PBS^-Ca2+Mg2+^, dried and suspended in 20% methanol. The absorbance was measured at λ=570 nm in a computer-interfaced, 96-well microtiter plate reader Multiskan RC photometer.

### Migration assay

#### Migration chamber preparation

Fibronectin assay: 8-μm insert membranes (Falcon BD Biosciences, USA) were sterilely covered with fibronectin (100 μg/ml, Falcon BD Biosciences). Both sides of the membrane were covered with 20 μl of the fibronectin suspension and incubated for 30 min at 37°C. Fibronectin was removed and the inserts were washed three times with sterile water. Subsequently, both sides of the membrane were immersed in a 0.1% albumin solution and incubated for 15 min. The inserts were washed three times with sterile water and dried. The prepared inserts were not stored, but used immediately after preparation.

#### Migration assay

The siRNA M2-transfected, negative siRNA-transfected and non-treated B16 cells were suspended in DMEM with no FBS, and applied to the upper section of the migration chamber, with 2.9×10^5^ cells/insert. Culture medium supplemented with 10% FBS applied to the lower section served as chemoattractant.

The migration was carried out at 37°C with 5% CO_2_. The time of migration was initially optimised and for B16 cells was 2 h. Thereafter (following the manufacturer’s instructions), the cells from the upper side of the membrane were removed with a cotton swab. The cells on the bottom side of the membrane were fixed and stained with a Diff-Quick set (Medion Diagnostics, Düdingen, Switzerland) and counted by light microscopy. The number of cells per membrane was determined, accumulated into groups, and the average was presented.

#### Metastasis assay

Eight- to twelve-week-old C57BL/6/IiW female mice were purchased from Maria Skłodowska-Curie Memorial Cancer Center and Institute of Oncology, Warsaw (Poland) and kept under specific pathogen-free (SPF) conditions. All experiments were performed under standard laboratory conditions according to Interdisciplinary Principles and Guidelines for the Use of Animals in Research, Marketing and Education issued by the New York Academy of Science Ad Hoc Committee on Animal Research and were approved by the 1st Local Committee for Experiments with the Use of Laboratory Animals, Wroclaw, Poland.

Mice were inoculated intravenously (i.v.) with 3×10^5^ B16 cells (collected from *in vitro* culture) in 0.2 ml of Hank’s medium into the lateral tail vein. Mice were sacrificed by cervical dislocation (21 days after cells inoculation). Lungs were excited and weighed immediately, and lung metastatic foci were counted.

## Results

### Inhibition of integrin β3 synthesis by RNA interference in vitro

**Figure 1:**
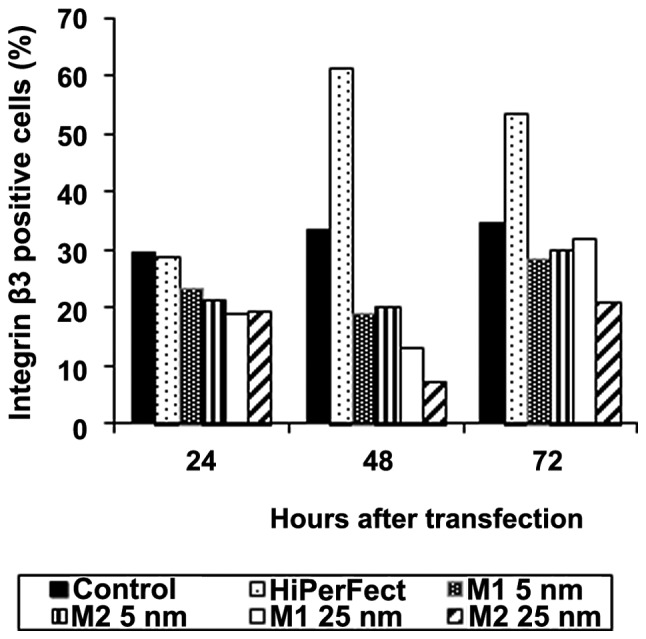
Time-course of siRNA-mediated inhibition of integrin β3 expression on B16 mouse melanoma cells. Cells were transfected with two different siRNA sequences (M1 and M2) at two concentrations and the protein expression was measured by FACS analysis. Non-transfected cells and cells treated only with transfection reagent served as controls.

B16 cells were transfected with 5 or 25 nM of M1 and M2 siRNAs. The expression of integrin β3 was measured by cytofluorometry after 24, 48 and 72 h after transfection. Both siRNA sequences led to the reduction of integrin β3 expression as compared to control, non-transfected cells; however, the sequence M2 appeared to be more potent. In both cases, the silencing effect increased with siRNA concentration. However, we also showed that the most effective concentration of siRNA was 25 nM and further increase in siRNA amount did not enhance the effect (data not shown). Moreover, our experiments confirmed that siRNA-mediated silencing of integrin β3 expression is transitory, with a highest inhibition of protein expression after 48 h after transfection. We observed almost 80% reduction of integrin β3 expression on B16 cells 48 h after transfection with M2 siRNA compared to untreated cells (Fig. 1).

None of the tested sequences showed cytotoxicity. The inhibition of B16 cells proliferation reached only 5%, 24 h after transfection as compared to the control, non-transfected cells, irrespective of siRNA sequence and concentration applied. B16 cells treated with siRNAs achieved a control proliferation rate 72 h after transfection.

**Figure 2:**
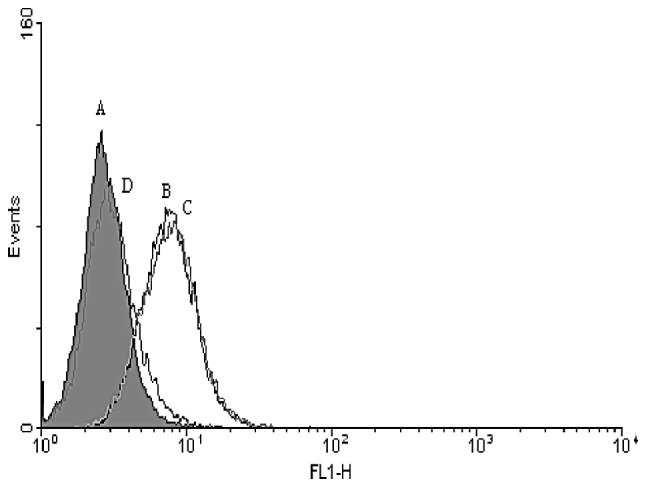
siRNA-mediated inhibition of integrin β3 expression on B16 melanoma cells. Non-transfected cells incubated with PBS (A) and McAb (B) served as controls. Cells were transfected with a negative, ‘blind’ siRNA (C) and siRNA against integrin β3 (D). The experiment was repeated 14 times and the histogram shows representative data.

Taking the above-mentioned results into account, we chose M2 sequence for further studies. Comparing the efficacy of integrin β3 silencing by a single and a double transfection, we found that it is possible to obtain a significant increase in the inhibition of integrin β3 expression due to a transfection repeated after additional 48 h. In that case, the inhibition of integrin β3 expression on B16 cells could reach even 98% (mean inhibition was 87±8%, which corresponded to 48±11% drop in the mean fluorescence canal values). Cells restored integrin β3 expression after 96 h after the first transfection. For negative siRNA, with no homology to any known mRNA, we showed a slight (5%) and insignificant increase in the integrin β3 expression. The representative histogram of transfected B16 cells is shown in Fig. 2.

**Figure 3:**
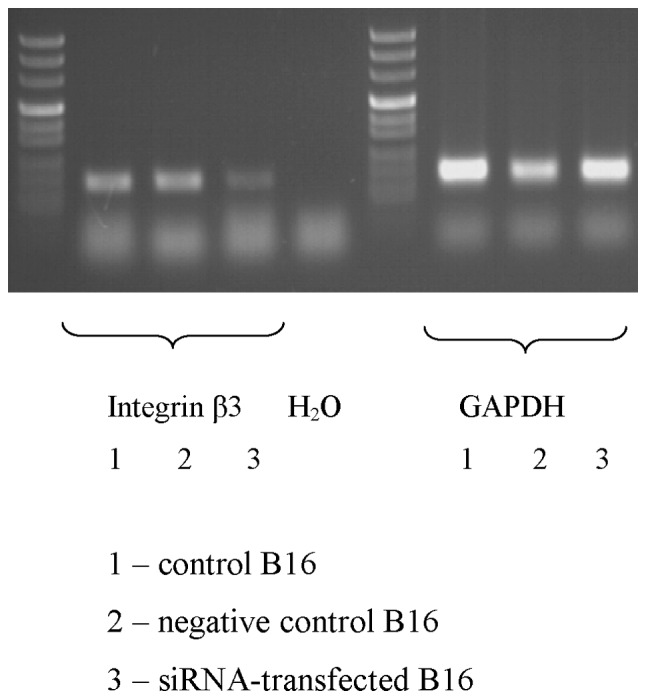
Changes in the expression of mRNA for integrin β3 in B16 cells transfected with siRNA for integrin β3 (1). Cells were transfected twice with 25 nM of siRNA. Untreated or transfected with negative siRNA B16 cells served as controls. Products of semi-quantitative PCR were dissolved in 1.7% agarose gel with ethidium bromide.

These changes were confirmed on mRNA level. Semi-quantitative PCR revealed a marked decrease in the expression of mRNA for integrin β3 as a result of the siRNA transfection. No significant differences were observed in the expression of integrin β3 mRNA between control, untreated cells and cells transfected with negative siRNA (Fig. 3).

### Inhibition of cell adhesion to matrix proteins by RNA interference in vitro

**Figure 4:**
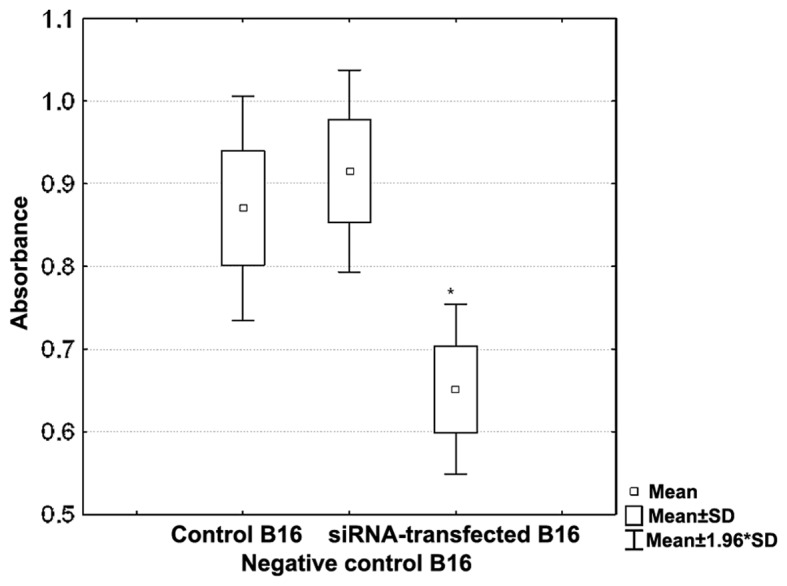
Inhibition of the interactions between B16 melanoma cells and fibrinogen by RNA interference. Cells were transfected twice with 25 nM of siRNA. The experiment was repeated twice and the histogram shows representative data. Values are means after subtraction of the absorbance of BSA and fibrinogen-coated wells ± SD. ^*^p<0.01 assessed with Kruskal-Wallis ANOVA.

To estimate the possible effects of integrin β3 silencing on the cell-ECM interactions, we studied adhesive properties of B16 cells on fibrinogen-coated plates. B16 cells were transfected with 25 nM of siRNA and the transfection was repeated after 48 h. The expression of integrin β3 was measured by cytofluorometry after additional 24 h. The experiment was repeated twice and in each attempt almost 90% silencing of integrin β3 was obtained. It corresponded to a statistically significant impairment of the adhesion to fibrinogen. siRNA-transfected B16 cells bound to fibrinogen-coated wells were 31% weaker in comparison to the control, non-transfected cells (Fig. 4).

### Inhibition of cell migration by RNA interference in vitro

**Figure 5:**
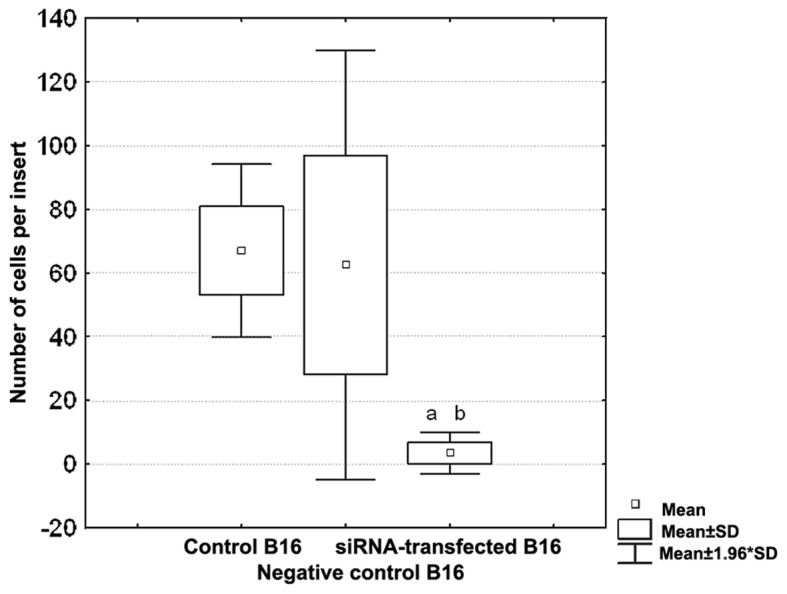
Inhibition of the migration through fibronectin-covered inserts of B16 cells transfected with siRNA against integrin β3. Cells were transfected twice with 25 nM of siRNA. Untreated B16 cells served as the control. Cells were applied to the upper section of the migration chamber at the density of 2.9×10^5^ cells/insert. The number of cells per membrane was determined, accumulated into groups, and the average is presented. Values are the mean ± SD. n=6–7, (a) p<0.01 vs. control B16, (b) p<0.05 vs. negative control B16 assessed with Kruskal-Wallis ANOVA.

To verify the influence of integrin β3 on the motility of B16 melanoma cells, the migration assay was performed. B16 cells with silenced expression of integrin β3 (80% lower than the control, untreated cells) were applied. Inhibition of integrin β3 expression caused almost complete impairment of the ability of B16 cells to migrate through the fibronectin-coated inserts (Fig. 5). Mean number of B16 siRNA-transfected cells detected on the bottom side of the membrane was 4±3, whereas this value for the control, untreated cells was 67±14 (p<0.01). No influence of the transfection with negative siRNA on the cell motility was observed (63±34).

### Inhibition of metastatic potential by RNA interference

**Figure 6:**
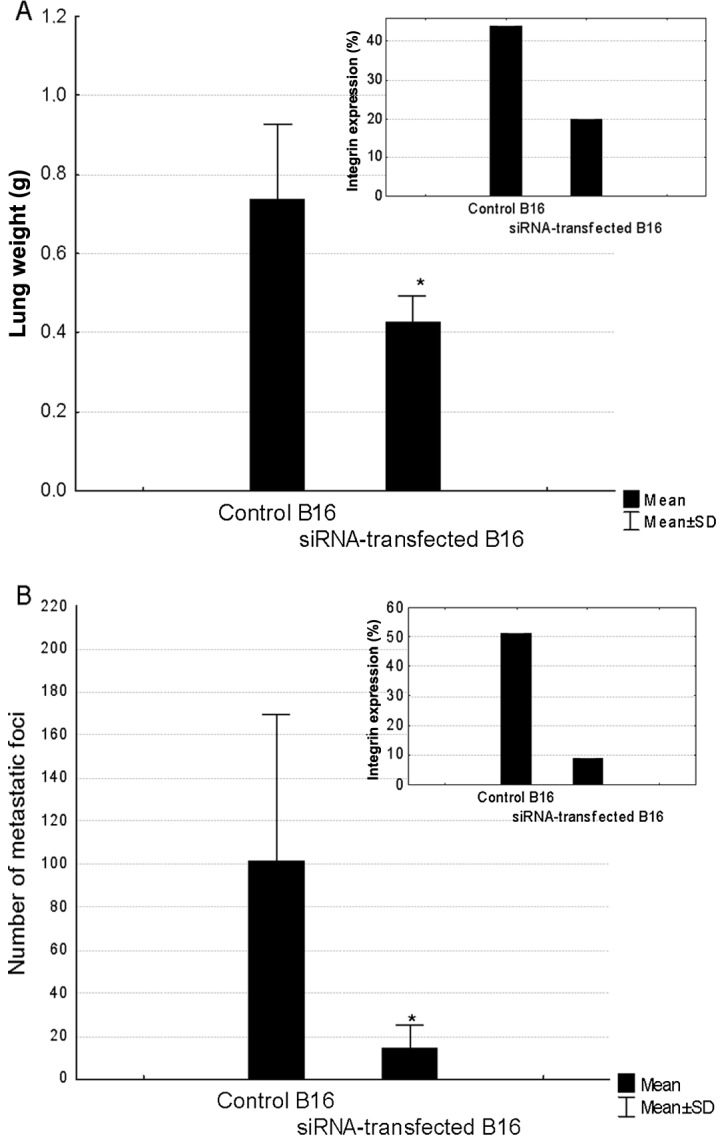
Relationship between integrin expression and metastatic potential of B16 cells. Cells were transfected once (A) or twice (B) with 25 nM of siRNA for integrin β3. Mice were inoculated intravenously with 3×10^5^ cells. Twenty-one days after inoculation of B16 cells, lungs were excised and weighed immediately, and lung metastatic foci were counted. Values are mean ± SD. n=7 (A) or n=8 (B), ^*^p<0.01 assessed with Kruskal-Wallis ANOVA.

C57/BL6 mice were inoculated intravenously (i.v.) with B16 cells transfected with anti-integrin β3 siRNA, negative siRNA or non-transfected, control ones. A correlation between the level of silencing of integrin β3 expression and the inhibition of metastatic potential of B16 cells was observed. In the first experiment, the expression of integrin β3 on siRNA-treated cells was inhibited by 55%. At the end of the experiment, the lungs were excised and weighed. The mean lung weight in the control mice was 0.74 g. It was significantly decreased in the group of mice inoculated with B16 cells transfected with siRNA against integrin β3 (Fig. 6A). The 42% drop in the lung weight in these mice corresponded to 55% decrease in the expression of CD61 in the transfected cells measured by cytofluorymetry prior to the melanoma cells inoculation. However, 83% silencing of integrin β3 expression led to 86% drop in the number of lung metastatic foci as compared to the control values (Fig. 6B). No significant inhibition of metastatic potential of B16 cells treated with negative siRNA was observed.

## Discussion

Many studies have shown that the expression of integrins alters frequently during malignant transformation. These changes comprise both alterations in the number and identity of integrin receptors on cancer cells (8). Special attention is focused on the role of both αvβ3 and αIIbβ3 in tumour growth, invasion and metastasis. Tumour cells expressing αvβ3 and/or αIIbβ3 display increased survival and growth *in vivo*(3), and increased metastatic potential (19). Upregulation of integrin expression results in alteration of the ability of malignant cells to interact with the extracellular matrix, and promotes migration as well as facilitates survival outside the tumour microenvironment.

The importance of both αvβ3 and αIIbβ3 has been extensively studied in melanoma. Presence of β3 subunit is a characteristic of melanoma, and is strongly associated with the disease progression and poor prognosis (1–2,20).

Integrins have been shown to be potential targets for drug development for therapeutic applications including anticancer treatment (21). Biological methods targeting integrins include monoclonal antibodies (16,22,23), peptides containing RGD or KGD motifs (24,25), RGD analogues (26), and more recently, siRNAs (27,28). RNA interference (RNAi) is a sequence-specific post-transcriptional gene silencing by double-stranded RNA. This mechanism, first discovered by Mello and Fire in *Caenorhabditis elegans* is present and conserved in a range of organisms (29). Despite the endogenous origin, siRNA can be introduced efficiently into the cells. For over a decade now, siRNAs have been successfully used for targeting and knockdown of sequence-specific mRNAs and has become a key experimental tool for the analysis of gene function. SiRNA have also moved into the clinic; several siRNA-based therapeutic strategies have entered clinical trials (30).

Herein, we report for the first time that siRNA can selectively and efficiently silence the expression of integrin β3 subunit in B16 melanoma cells. The effect is manifested 48 h after transfection and can be significantly enhanced by double transfection (first, shortly after seeding of the cells and second, 48 h later). Integrin β3-silencing does not affect the proliferation rate of B16 cells.

Clinically, metastatic phenotype of melanoma tumours depends on peculiar adhesive, invasive and migratory properties of tumour cells. This is mostly correlated with the expression of the adhesion receptor integrin αvβ3 and αIIbβ3.

In order to metastasise, tumour cells need to detach from the primary tumour, gain access to blood vessels, survive in blood stream, then attach to vascular endothelial cells, extravasate from blood vessels and finally, colonize distant tissues and organs. These steps are strongly dependent on the cross-talk between tumour and endothelial cells as well as on cell-ECM interactions. Among ECM ligands for β3 integrins, fibrinogen, fibronectin and vitronectin are of special importance (31–34). It has been shown that in fibrinogen-deficient mice, a significant reduction in the number of lung metastases formed by B16 melanoma and LLC (Lewis Lung Carcinoma) cells was observed (35). Proteolytic fragments or recombinant peptides containing certain domains of fibronectin can inhibit integrin-mediated adhesion, angiogenesis and metastasis in various experimental tumour models (36–39; reviewed in refs. 21,41). In our studies, the transfection of B16 melanoma cells with siRNA for integrin β3, resulting in 90% silencing of protein expression, corresponding to a statistically significant impairment of the adhesion to fibrinogen. siRNA-transfected B16 cells bound to fibrinogen-coated plates were 31% weaker than the control, integrin-positive cells. These observations probably point toward the involvement of other adhesive proteins in the interactions between B16 melanoma cell and fibrinogen. These may include α4β1 or α5β1 integrins (40,41).

In these studies, we also show that siRNA-mediated silencing of integrin β3 expression significantly affects the metastatic potential of B16 cells. B16 cells that express lower levels of integrin β3 form less metastatic foci in lungs when injected into tail vein in comparison to the control non-transfected cells. This may result from the impairment of several steps which are crucial for the colonization of distant organs, i.e. i) survival in bloodstream, ii) attachment to vascular endothelial cells, iii) basal membrane disintegration, iv) extravasation from vessel lumen, and v) establishment of secondary tumours. Integrin β3 expressed on the surface of B16 cells is involved in all these steps. Since the integrin β3-knockdown is transitory, it seems that the impairment of early steps of this ‘metastatic cascade’ is crucial for long-term effects observed in our studies. It has been shown that the survival rate of tumour cells in bloodstream may be connected with the interactions between tumour cells and platelets, which, in turn, seem to be fibrinogen related. Recent studies have demonstrated that platelets and fibrinogen facilitate each other in protecting tumour cells from natural killer cytotoxicity (42). It has also been suggested that the formation of platelet-fibrin-tumour cell aggregates may be causally related to endothelial adhesion and metastatic potential (43–45). Since the adhesion to fibrinogen is inhibited in β3-deficient cells, this may explain the low metastatic potential of siRNA-transfected B16 cells.

β3-silenced cells are probably unable to adhere to vessel walls. It may be suggested that the production and/or activation of matrix metalloproteinases (MMPs) essential for basement membrane disruption is inhibited (46,47). This may clearly affect the migration of B16 cells through the vessel walls. We also show that silencing of β3 expression in B16 cells leads to a dramatic loss of migratory properties. This could be explained both by the inhibition of B16 cells-ECM interactions as well as by the abrogation of signal transduction pathways promoting cell motility (48–51).

In summary, our experiments have proved that siRNA transfection is an effective tool for the silencing of integrin β3 expression in B16 melanoma cells. The inhibition of integrin β3 expression on cell surface is correlated with impaired motility, ability to bind to ECM proteins and significantly lower metastatic potential. Furthermore, our studies suggest that the impairment of early steps of this ‘metastatic cascade’ is crucial for long-term effects.

## References

[1] Albelda SM, Mette SA, Elder DE, Stewart R, Damjanovich L, Herlyn M, Buck CA (1990). Integrin distribution in malignant melanoma: association of the β3 subunit with tumor progression. Cancer Res.

[2] Hieken TJ, Farolan M, Ronan SG, Shilkaitis A, Wild L, Das Gupta TK (1996). β3 integrin expression in melanoma predicts subsequent metastasis. J Surg Res.

[3] Trikha M, Timar J, Zacharek A, Nemeth JA, Cai Y, Dome B, Somlai B, Raso E, Ladanyi A, Honn KV (2002). Role for β3 integrins in human melanoma growth and survival. Int J Cancer.

[4] Cooper CR, Chay CH, Pienta KJ (2002). The Role of alpha(v)beta(3) in prostate cancer progression. Neolplasia.

[5] Rezaeipoor R, Chaney EJ, Oldenburg AL, Boopart S (2009). Expression order of alpha-v and beta-3 integrin subunits in the N-methyl-N-nitrosourea-induced rat mammary tumor model. Cancer Invest.

[6] Leu SJ, Lam SC, Lau LF (2002). Pro-angiogenic activities of CYR61 (CCN1) mediated through integrins avb3 and a6b1 in human umbilical vein endothelial cells. J Biol Chem.

[7] Nam JO, Kim JE, Jeong HW, Lee SJ, Lee BH, Choi JY, Park RW, Park JY, Kim IS (2003). Identification of the αvβ3 integrin-interacting motif of βig-h3 and its anti-angiogenic effect. J Biol Chem.

[8] Mizejewski GJ (1999). Role of integrins in cancer: survey of expression patterns. Proc Soc Exp Biol Med.

[9] Minamiguchi K, Kumagai H, Masuda T, Kawada M, Ishizuka M, Takeuchi T (2001). Thiolutin, an inhibitor of HUVEC adhesion to vitronectin, reduces paxillin in HUVECs and suppresses tumour cell-induced angiogenesis. Int J Cancer.

[10] Nikos E, Tsopanoglou NE, Andriopoulou P, Maragoudakis ME (2002). On the mechanism of thrombin-induced angiogenesis: involvement of αvβ3-integrin. Am J Physiol Cell Physiol.

[11] Burns GF, Cosgrove L, Triglia T, Beall JA, Lopez AF, Werkmeister JA, Begley CG, Haddad AP, d’Apice AJ, Vadas MA (1986). The IIb-IIIa glycoprotein complex that mediates platelet aggregation is directly implicated in leukocyte adhesion. Cell.

[12] Timar J, Trikha M, Szekeres K, Bazaz R, Tovari J, Silletti S, Raz A, Honn KV (1996). Autocrine motility factor signals integrin-mediated metastatic melanoma cell adhesion and invasion. Cancer Res.

[13] Oleksowicz L, Mrowiec Z, Schwartz E, Khorshidi M, Dutcher J, Puszkin E (1995). Characterization of tumour-induced platelet aggregation: the role of immunorelated GPIb and GPIIb/IIIa expression by MCF-7 breast cancer cells. Thromb Res.

[14] Sheu JR, Lin CH, Peng HC, Huang TF (1994). Triflavin, an Arg-Gly-Asp-containing peptide, inhibits human cervical carcinoma (HeLa) cell-substratum adhesion through an RGD-dependent mechanism. Peptides.

[15] Yun Z, Menter DG, Nicolson GL (1996). Involvement of integrin alphavbeta3 in cell adhesion, motility, and liver metastasis of murine RAW117 large cell lymphoma. Cancer Res.

[16] Cohen SA, Trikha M, Mascelli MA (2000). Potential future clinical applications for the GPIIb/IIIa antagonist, abciximab in thrombosis, vascular and oncological indications. Pathol Oncol Res.

[17] Auzzas L, Zanardi F, Battistini L, Burreddu P, Carta P, Rassu G, Curti C, Casiraghi G (2010). Targeting alphavbeta3 integrin: design and applications of mono- and multifunctional RGD-based peptides and semipeptides. Curr Med Chem.

[18] Skehan P, Storeng R, Sudiero D, Monks A, McMahon J, Vistica D, Warren JT, Bokesch H, Kenney S, Boyd MR (1990). New colorimetric cytotoxicity assay for anticancer-drug screening. J Natl Cancer Inst.

[19] Chang YS, Chen YQ, Timar J, Nelson KK, Grossi IM, Fitzgerald LA, Diglio CA, Honn KV (1992). Increased expression of alpha IIb beta 3 integrin in subpopulations of murine melanoma cells with high lung-colonizing ability. Int J Cancer.

[20] Van Belle PA, Elenitsas R, Satyamoorthy K, Wolfe JT, Guerry DI, Schuchter L, Van Belle TJ, Albelda S, Tahin P, Herlyn M, Elder DE (1999). Progression-related expression of beta3 integrin in melanomas and nevi. Hum Pathol.

[21] Perdih A, Dolenc MS (2010). Small molecule antagonists of integrin receptors. Curr Med Chem.

[22] Brooks PC, Stromblad S, Klemke R, Visscher D, Sarkar FH, Cheresh DA (1995). Antiintegrin alpha v beta 3 blocks human breast cancer growth and angiogenesis in human skin. J Clin Invest.

[23] Mitjans F, Meyer T, Fittschen C, Goodman S, Jonczyk A, Marshall JF, Reyes G, Piulats J (2000). In vivo therapy of malignant melanoma by means of antagonists of αv integrins. Int J Cancer.

[24] Isoai A, Ueno Y, Giga-Hama Y, Goto H, Kumagai HA (1992). A novel Arg-Gly-Asp containing peptide specific for platelet aggregation and its effect on tumor metastasis: a possible mechanism of RGD peptide-mediated inhibition of tumor metastasis. Cancer Lett.

[25] Buerkle MA, Pahernik SA, Sutter A, Jonczyk AKM, Dellian M (2002). Inhibition of the alpha-nu integrins with a cyclic RGD peptide impairs angiogenesis, growth and metastasis of solid tumours in vivo. Br J Cancer.

[26] Perkins JJ, Duong LT, Fernandez-Metzler C, Hartman GD, Kimmel DB, Leu C-T, Lynch JJ, Prueksaritanont T, Rodan GA, Rodan SB (2003). Non-peptide alpha(v)beta(3) antagonists: identification of potent, chain-shortened RGD mimetics that incorporate a central pyrrolidinone constraint. Bioorg Med Chem Lett.

[27] Han HD, Mangala LS, Lee JW, Shahzad MM, Kim HS, Shen D, Nam EJ, Mora EM, Stone RL, Lu C (2010). Targeted gene silencing using RGD-labeled chitosan nanoparticles. Clin Cancer Res.

[28] Roman J, Ritzenthaler JD, Roser-Page S, Sunx, Han S (2010). Alpha5beta1-integrin expression is essential for tumor progression in experimental lung cancer. Am J Respir Cell Mol Biol.

[29] Fire A, Xu S, Montgomery MK, Kostas SA, Driver SE, Mello CC (1998). Potent and specific genetic interference by double-stranded RNA in *Caenorhabditis elegans*. Nature.

[30] Castanotto D, Rossi JJ (2009). The promises and pitfalls of RNA-interference-based therapeutics. Nature.

[31] Hart IR, Birch M, Marshall JF (1991). Cell adhesion receptor expression during melanoma progression and metastasis. Cancer Metastasis Rev.

[32] Seftor RE (1998). Role of the beta3 integrin subunit in human primary melanoma progression: multifunctional activities associated with alpha(v)beta3 integrin expression. Am J Pathol.

[33] Switala-Jelen K, Dabrowska K, Opolski A, Lipinska L, Nowaczyk M, Gorski A (2004). The biological functions of beta3 integrins. Folia Biol.

[34] Akiyama SK, Olden K, Yamada KM (1995). Fibronectin and integrins in invasion and metastsis. Cancer Metastasis Rev.

[35] Palumbo JS, Kombrinck KW, Drew AF, Grimes TS, Kiser JH, Degen JL, Bugge TH (2000). Fibrinogen is an important determinant of the metastatic potential of circulating tumor cells. Blood.

[36] Yi M, Ruoslahti E (2001). A fibronectin fragment inhibits tumor growth, angiogenesis and metastasis. Proc Natl Acad Sci USA.

[37] Gong W, Liu Y, Huang B, Lei Z, Wu F-H, Li D, Feng Z-H, Zhang G-M (2008). Recombinant CBD-HepII polypeptide of fibronectin inhibits αvβ3 signaling and hematogenous metastasis of tumor. Biochem Biophys Res Commun.

[38] Ramos OH, Kauskot A, Cominetti MR, Bechyne I, Salla Pontes CL, Chareyre F, Manent J, Vassy R, Giovannini M, Legrand C (2008). A novel avb3-blocking disintegrin containing the RGD motive DisBa-01, inhibits bFGF-induced angiogenesis and melanoma metastasis. Clin Exp Metastasis.

[39] Sheldrake HM, Patterson LH (2009). Function and antagonism of beta3 integrins in the development of cancer therapy. Curr Cancer Drug Targets.

[40] Gehlsen KR, Davis GE, Sriramarao P (1992). Integrin expression in human melanoma cells with differing invasive and metastatic properties. Clin Exp Metastasis.

[41] Rebhun RB, Cheng H, Gershenwald JE, Fan D, Fidler IJ, Langley RR (2010). Constitutive expression of the alpha4 integrin correlates with tumorigenicity and lymph node metastasis of the B16 murine melanoma. Neoplasia.

[42] Zheng S, Shen J, Jiao Y, Zhang C, Wei M, Hao S, Zeng X (2009). Platelets and fibrinogen facilitate each other in protecting tumor cells from natural killer cytotoxicity. Cancer Sci.

[43] Cavanaugh PG, Sloane BF, Honn KV (1988). Role of the coagulation system in tumor-cell-induced platelet aggregation and metastasis. Haemostasis.

[44] Crissman JD, Hatfield JS, Menter DG, Sloane B, Honn KV (1988). Morphological study of the interaction of intravascular tumor cells with endothelial cells and subendothelial matrix. Cancer Res.

[45] Liu Y, Zhao F, Gu W, Yang H, Meng Q, Zhang Y, Yang H, Duan Q (2009). The roles of platelet GPIIb/IIIa and alphavbeta3 integrins during HeLa cells adhesion, migration, and invasion to monolayer endothelium under static and dynamic shear flow. J Biomed Biotechnol.

[46] Ria R, Vacca A, Ribatti D, Di Raimondo F, Merchionne F, Dammacco F (2002). Alpha(v)beta(3) integrin engagement enhances cell invasiveness in human multiple myeloma. Haematologica.

[47] Sato T, Sakai T, Noguchi Y, Takita M, Hirakawa S, Ito A (2004). Tumor-stromal cell contact promotes invasion of human uterine cervical carcinoma cells by augmenting the expression and activation of stromal matrix metalloproteinases. Gynecol Oncol.

[48] Cary LA, Guan JL (1999). Focal adhesion kinase in integrin-mediated signaling. Front Biosci.

[49] Hao H, Naomoto Y, Bao X, Watanabe N, Sakurama K, Noma K, Motoki T, Tomono Y, Fukazawa T, Shirakawa Y (2009). Focal adhesion kinase as potential target for cancer therapy (Review). Oncol Rep.

[50] Yang J, Price MA, Li GY, Bar-Eli M, Salgia R, Jagedeeswaran R, Carlson JH, Ferrone S, Turley EA, McCarthy JB (2009). Melanoma proteoglycan modifies gene expression to stimulate tumor cell motility, growth, and epithelial-to-mesenchymal transition. Cancer Res.

[51] Hou CH, Yang RS, Hou SM, Tang CH (2011). TNF-α increases αvβ3 integrin expression and migration in human chondrosarcoma cells. J Cell Physiol.

